# Clinico-radiological analysis and outcomes of management of thalamic space-occupying lesions: A three-year retrospective case series

**DOI:** 10.12669/pjms.41.13(PINS-NNOS).13441

**Published:** 2025-12

**Authors:** Haseeb Mehmood Qadri, Zia Ul Rehman Najeeb, Nasruddin Ansari, Shahrukh Rizvi, Ahtesham Khizar

**Affiliations:** 1 Haseeb Mehmood Qadri, MBBS. Punjab Institute of Neurosciences, Lahore, Pakistan; 2 Zia-Ul-Rehman Najeeb, MBBS. Punjab Institute of Neurosciences, Lahore, Pakistan; 3 Nasruddin Ansari, MBBS. Punjab Institute of Neurosciences, Lahore, Pakistan; 4 Shahrukh Rizvi, MBBS, FCPS. Punjab Institute of Neurosciences, Lahore, Pakistan; 5 Ahtesham Khizar, MBBS, FCPS. Punjab Institute of Neurosciences, Lahore, Pakistan

**Keywords:** Biopsy, Brain Neoplasms, Magnetic Resonance Imaging, Neuronavigation, Pakistan, Thalamic Neoplasms, Treatment Outcome

## Abstract

**Background and Objective:**

Thalamic tumors are rare, deep-seated tumors with a paucity of literature globally, and yet no existing literature based on the Pakistani population. The objective of this study was to evaluate the clinical presentations and radiological findings of thalamic space-occupying lesions (SOLs) and analyze the outcomes of management practices.

**Methodology:**

A bi-departmental retrospective observational case series of 13 patients who underwent surgical excision or cerebrospinal fluid (CSF) diversion for thalamic SOLs at the Punjab Institute of Neurosciences (PINS) Hospital from 1^st^ January 2022 to 31^st^ December 2024. A descriptive analysis was done for clinical-radiological and outcome-based variables.

**Results:**

The cohort comprises 33% (4) pediatric patients (mean age, 8.75±6.07 years) and 67% (9) adults (mean age, 33.67±14.82 years), with a male predilection of 56.3% (9). The most common presentation was focal neurological deficit in 77% (10). Magnetic Resonance Imaging (MRI) revealed that tumors were hypointense on T1-weighted images (T1WI) in 77% (10) of patients and hyperintense on T2-weighted images (T2WI) in 92% (12), with 77% (10) of them being contrast enhancing. Ventriculoperitoneal Shunt (VPS) was required in 15% (2) of patients, improvement of Glasgow Coma Scale (GCS) was in 15% (2), and Karnofsky Performance Status (KPS) after surgery was improved in 23% (3). Glioblastoma 61.5% (8) was the most common diagnosis on histopathology.

**Conclusion:**

High-grade thalamic gliomas usually affect adult males and present with focal neurological symptoms in the Pakistani population, requiring MRI with Gadolinium contrast evaluation for the diagnosis. Neuronavigation-guided (NNG) biopsy is the common modality of neurosurgical intervention, with minimal improvement, and glioblastoma, the common histopathological diagnosis for thalamic tumors.

## INTRODUCTION

Space-occupying lesions (SOLs) of the thalamus, especially gliomas, pose substantial therapeutic problems because of their intricate anatomy and deep location. Thalamic tumors comprise 1% of all brain tumors, 5% of all pediatric intracranial tumors, and 15% of malignant pediatric tumors.^1^ The epidemiology of thalamic tumors indicates a predominance in younger populations, particularly children, with a mean age of 8.9 years at presentation and a male : female ratio of 1.5.^2^ Thalamic neoplasms can be unilateral (low-grade astrocytomas), thalamopeduncular tumors (benign pilocytic astrocytomas), bilateral (high-grade astrocytomas), oligodendrogliomas, or gangliogliomas.^3^ Thalamic gliomas in adults are glioblastomas with a median age of 45 years at presentation and have a poor prognosis if associated with the H3K27M mutant.^4^

These tumors can manifest with a variety of symptoms, including motor and sensory deficits, psychological disturbances, and hydrocephalus, which complicate diagnosis and management.^5^ Radiologically, thalamic tumors exhibit distinct features on magnetic resonance imaging (MRI) with contrast (modality of choice) with SWI (Susceptibility Weighted Imaging), MRS (Magnetic Resonance Spectroscopy), and DTI (Diffuse Tensor Imaging). These tumors typically present as well-defined masses with varying degrees of enhancement and edema.^6^ Surgical resection remains the primary treatment modality, with gross total resection (GTR) linked to improved surgical rates, although complete resection is not always feasible due to tumor proximity to critical brain structures. Advances in surgical techniques, including neuronavigation guidance (NNG) and intraoperative monitoring of motor and somatosensory evoked potentials, transcortical (usually) and interhemispheric approaches (sporadically), yield better outcomes.^7^ Adjuvant treatment recommendations include radiotherapy, chemotherapy, and targeted therapies for thalamic tumors that cannot be operated on, depending upon recurrence or residual disease, histopathology, molecular markers, patient health status, and the goal of the treatment. Palliative care for irresectable tumors focuses on symptomatic treatment, supportive care, and improving quality of life.^8^

Despite the existing literature, gaps remain in understanding the comprehensive management and long-term outcomes of thalamic tumors, particularly within the Pakistani context. This is the first Pakistani study to address these gaps by providing a detailed and comprehensive analysis of clinical-radiological presentations and elaborating on the management of thalamic tumors at the highest-volume center in Pakistan. By doing so, it seeks to contribute valuable insights into the epidemiology and treatment of these challenging tumors, ultimately enhancing patient care and outcomes in this demographic. This study aimed to characterize the demographic and clinical presentations of thalamic space-occupying lesions, describe radiological features on MRI, analyze surgical management approaches, and evaluate short-term neurological outcomes following surgical intervention at the Punjab Institute of Neurosciences (PINS) Hospital.

## METHODOLOGY

A retrospective observational case series was conducted at the Departments of Neurosurgery, Unit-I and Unit-III, Punjab Institute of Neurosciences Hospital, Pakistan, from 1^st^ January 2022 to 31^st^ December 2024. This study included all the patients who underwent surgical excision or supportive procedures like cerebrospinal fluid (CSF) diversion and biopsy for thalamic SOLs. A total of 13 patients were identified through hospital records and included in this bi-departmental, non-probability consecutive case series. Non-probability consecutive sampling was employed due to the rare nature of thalamic tumors and the specialized tertiary care setting, ensuring inclusion of all eligible cases during the study period.

This study was reviewed and approved by the Institutional Review Board of our institution with reference no. 2034/IRB/PINS/Approval/2025, dated January 23, 2025.

### Inclusion criteria:

- Patients of any age or gender with a radiologically confirmed thalamic lesion (primary involvement).
- Patients who underwent neurosurgical intervention at our center, including tumor excision and biopsy, and CSF diversion procedures like ventriculoperitoneal shunt (VPS) or endoscopic third ventriculostomy (ETV).
- Availability of pre-operative imaging, operative records, and post-operative follow-up data and histopathological confirmation of diagnosis (where applicable).
- Minimum three months follow-up data available post-surgery.

### Exclusion Criteria

- The lesion was secondary, involving the thalamus from tumors primarily located elsewhere.
- Patients who were managed non-surgically or conservatively.
- Incomplete clinical or imaging records make analysis unreliable.
- Patients who were lost to follow-up immediately after surgery or before outcome assessment.
- Cases with coexisting multifocal intracranial pathology where thalamic SOLs could not be independently evaluated.

Data was collected from patients’ medical records and Picture Archiving and Communication System (PACS). This included patient files, radiological data, operative notes, clinical notes, post-operative stay in the hospital, post-operative clinic follow-up, and rehabilitation/recovery. A structured questionnaire was designed on Google Forms to collect data. The variables included: demographic variables (age, gender), clinical presentations (symptom duration, neurological examination findings, GCS, KPS), radiological parameters (lesion location, MRI signal characteristics, enhancement patterns), surgical details (approach, extent of resection, complications), and histopathological diagnoses. All imaging was reported by consultant neuroradiologists, while clinical assessments were conducted by final-year postgraduate residents well documented in the discharge certificate to ensure data quality and accuracy.

### Data analysis:

Given the small sample size (n=13) and descriptive nature of this case series, descriptive statistical methods were employed to analyze the clinical, radiological, and surgical variables of the included patients. Continuous variables, such as age, Glasgow Coma Score (GCS), and Karnofsky Performance Status (KPS), were presented as mean ± standard deviation. Categorical variables, including gender, location of the lesion, presenting symptoms, type of intervention, extent of resection, and histopathological diagnosis, were reported as frequencies and percentages.

Comparative analysis between pre- and post-operative GCS and KPS was performed to evaluate neurological outcomes and functional status following intervention. The extent of surgical resection was also assessed for changes in clinical status. Neuroimaging findings were described, including signal characteristics on T1-weighted images (T1WI), T2WI, and gadolinium contrast enhancement patterns on MRI. Statistical significance was not calculated due to the exploratory nature of this case series. Statistical significance was not calculated due to the exploratory nature of this case series.

## RESULTS

A total of 13 patients diagnosed with thalamic space-occupying lesions were included in the study. The cohort comprised 77% (10) males and 23% (3) females, with a male-to-female ratio of 3.3:1. In addition, pediatric patients (≤18 years) accounted for 33% (4), while 67% (9) of patients were adults. The mean age among pediatric patients was 8.75 ± 6.07 years, whereas the adult group had a mean age of 33.67 ± 14.82 years.

The most frequently encountered presenting complaint was focal neurological deficit in 67% (9) of patients, predominantly manifesting as hemiparesis or hemiplegia. In addition, headache was reported in 77% (10) of patients, followed by vomiting in 67% (9) of patients, and seizures occurred in 38.5% (5) of patients. Furthermore, 54% (7) of patients had signs of raised intracranial pressure, whereas neurocognitive disturbances were observed in 23% (3) of patients.

MRI revealed that 77% (10) of lesions were hypointense on T1-weighted imaging, while hyperintensity on T2-weighted sequences was observed in 92% (12). Post-contrast enhancement was noted in 77% (10) of patients. Cystic components were identified in 38.5% (5) of lesions. On radiology, preoperative MRI suggested glioma in 77% (10) of patients, with midline shift in 38.5% (5) of patients, and 15% (2) showed features of obstructive hydrocephalus due to mass effect on the third ventricle. Furthermore, lesion laterality was split to 38.5% (5) in the left side and 61.5% (8) to the right side of the thalamus, and 7.7% (1) exhibited bilateral thalamic involvement.

All our patients underwent neuronavigation-guided biopsy (NNG). However, NNG excision was feasible in only 38.5% (5) of patients, and partial resection was achieved in 31% (4) of patients. Additionally, cyst aspiration was attempted in a single case 7.7% (1), and near-total resection was only achieved in 7.7% (1) of patients. Preoperative CSF diversion with a ventriculoperitoneal shunt was needed in 15% (2) of patients, primarily for obstructive hydrocephalus.

On histopathology, the most frequent lesion was glioblastoma in 61.5% (8) of patients, followed by pilocytic astrocytoma in 31% (4) of patients and diffuse midline glioma with H3K27-mutant in 7.7% (1) of patients (Table-I).

**Table-I:** Demographic, Clinical, and Surgical Characteristics of 13 Patients with Thalamic Space-Occupying Lesions

Pt. #	Age (years)	Gender (M/F)	Site of Thalamic Lesion	Preoperative CSF diversion	Procedure Type	Type of Resection	Histopathology
1	6	M	B/L	-	NNG biopsy	-	Pilocytic astrocytoma
2	24	M	L	-	NNG biopsy	-	Diffuse midline glioma H3K27-altered
3	2	M	R	-	NNG excision + biopsy	PR	Pilocytic astrocytoma
4	34	F	L	-	NNG biopsy	-	Glioblastoma
5	11	M	L	-	NNG excision + biopsy	PR	Pilocytic astrocytoma
6	27	M	R	-	NNG biopsy + cyst aspiration	-	Glioblastoma
7	26	M	R	-	NNG biopsy	-	Glioblastoma
8	50	F	L	-	NNG biopsy	-	Glioblastoma
9	16	M	R	VPS	NNG biopsy	-	Pilocytic astrocytoma
10	30	M	L	-	NNG excision + biopsy	NR	Glioblastoma
11	66	F	R	-	NNG biopsy	-	Glioblastoma
12	25	M	R	VPS	NNG excision + biopsy	PR	Glioblastoma
13	21	M	R	-	NNG excision + biopsy	PR	Glioblastoma

M: Male, F: Female, B/L: Bilateral, R: Right, L: Left, VPS: Ventriculoperitoneal shunt, NNG-biopsy: Neuronavigation-guided biopsy, PR: Partial resection and NR: Near-total resection.

Preoperative GCS was 15 in 85.0% (11) of patients, 14 in 15% (2) of patients, and 10 in 7.7% (1) of patients. Postoperatively, GCS remained static in most of the patients; however, GCS improved in 15% (2) of patients and deteriorated in 7.7% (1) of patients. Similarly, mean KPS remained static in 30% (4), with only 23% (3) showing improvement and 46% (6) showing functional decline. Furthermore, 15% (2) developed new postoperative focal deficits, and rest of the patient did not show any further deterioration in their neurology (Table-II).

**Table-II:** Pre-operative and Post-operative Neurological Status of Patients with Thalamic Space-Occupying Lesions

Pt. #	Pre-operative Clinical Manifestations	Pre-operative GCS	Pre-operative KPS	Post-operative Clinical Manifestations	Post-operative GCS	Post-operative KPS
1	No focal deficit	14	60	Right-sided body weakness 3/5	15	60
2	Right-sided body weakness 4/5	15	70	Right-sided body weakness 4/5	15	70
3	Left-sided body weakness 3/5	15	70	Left-sided body weakness 3/5	15	70
4	Right-sided body weakness 4/5	15	80	Right-sided body weakness 4/5	15	80
5	Right-sided body weakness 3/5	15	90	Right-sided body weakness 3/5	15	70
6	Left-sided body weakness 3/5	15	100	Left-sided body weakness 3/5	15	70
7	Left-sided body weakness 3/5	15	100	Left-sided body weakness 3/5	15	80
8	Right-sided body weakness 3/5	15	100	Right-sided body weakness 3/5	15	80
9	No focal deficit	15	90	No focal deficit	15	100
10	Left-sided body weakness 3/5	15	30	Left-sided body weakness 3/5	12	40
11	Left-sided body weakness 3/5	14	60	Left-sided body weakness 3/5	14	50
12	No focal deficit	15	70	Right-sided body weakness 3/5	15	60
13	No focal deficit	10	10	No focal deficit	13	90

GCS: Glasgow Coma Scale, KPS: Karnofsky Performance Status Scale.

## DISCUSSION

### Demographics and clinical significance:

Thalamic space-occupying lesions represent a rare subset of intracranial tumors, comprising 0.84-5.2% of all brain tumors and 5% of pediatric intracranial SOLs.^1^,^3^ Our study represents the first dedicated analysis of thalamic tumors conducted in Pakistan, providing insights into the demographics, clinical presentation, radiological features, histopathology, and management strategies within limited resources.

The demographic profile in our cohort showed a notable adult predominance of 67% (9) vs. 33% (4) of pediatric, which differs from the typical pediatric predilection reported in Western literature. This finding may be attributed to the recent establishment of the pediatric neurosurgery division in our department rather than true epidemiological differences. The mean ages of 8.75±6.07 years (pediatric) and 33.67±14.82 years (adult) align with previous study by Palmisciano et al.^4^ The pronounced male predominance (77%) observed in our series is consistent with Sai Kiran and Cinalli’s study, supporting the established gender predilection for thalamic neoplasms.^9^,^10^

### Clinical presentation patterns:

The clinical manifestations in our cohort reflected the thalamus’s role as a critical relay station for sensory and motor pathways. Focal neurological deficits were the predominant presentation in 67% (9), primarily manifesting as hemiparesis or hemiplegia, which exceeded the 51.8% reported by Palmisciano et al.^4^ This higher incidence may indicate later presentation in our population or more aggressive tumor biology.

The constellation of symptoms—headache in 77% (10), vomiting in 67% (9), and seizures in 38% (5)—reflects the mass effect and intracranial hypertension commonly associated with deep-seated lesions. The preservation of Glasgow Coma Scale scores of 15 in 85% (11) of patients suggests relatively early presentation, which is encouraging for surgical outcomes. Even small thalamic lesions can significantly impact ventricular CSF circulation, explaining why 43.8% of patients presented with signs of raised intracranial pressure, which was similar to the findings of Sai Kiran’s study.^9^

### Radiological Characteristics and Diagnostic Implications:

MRI plays an important role in the diagnosis, lesion characterization, management planning, and follow-up of thalamic tumors. It also gives insight into the behavior of the lesion, helping to differentiate between high-grade and low-grade lesions.^11^ The predominant T1 hypointensity in 77% (10) and T2 hyperintensity in 92% (12) reflect the typical imaging signature of thalamic SOLs. Contrast enhancement was observed in 77% (10) of cases, higher than the 40% reported by Renedo et al., suggesting a higher proportion of high-grade lesions in our cohort.^2^,^6^

The right-sided predominance in 61.5% (8) and the presence of cystic components in 38.5% (5) are important surgical considerations. Hydrocephalus necessitating preoperative CSF diversion occurred in only 15% (2) of patients, indicating that most lesions were diagnosed before causing significant ventricular obstruction. These radiological patterns provide valuable guidance for surgical approach selection and prognostic assessment.

### Surgical Management Challenges and Outcomes:

In the past, thalamic lesions were considered inoperable because high mortality and morbidity followed surgery, and a conservative approach, including stereotactic biopsy (STB) and radiotherapy (RT), was preferred over a radical resection.^7^,^8^ However, current literature shows a better prognosis following radical resection in selected patients, especially those with pilocytic astrocytoma.^8^,^9^ Previously, stereotactic biopsy with a conservative approach was preferred; however, with improved surgical techniques, total or partial resection is now more frequently performed, particularly for noninfiltrating, low-grade tumors.^12^ In addition, microsurgery with postoperative adjuvant therapy can alleviate patients’ symptoms, improve quality of life, and increase survival of the patient.^13^ Lim et al. have reported that surgical resection of above 80% of the lesion has shown better outcomes and prognosis compared to biopsy.^14^

All patients in our series underwent neuronavigation-guided procedures, representing current standard of care for deep-seated lesions. However, the low rate of near-total resection of 7.7% (1) as compared to Palmisciano’s study, in which complete excision was achieved in 25% of patients and the predominance of biopsy-only procedures reflect limited surgical expertise in managing deep seated thalamic lesions.^4^

Partial resection was achieved in 31% (4) of cases, which, while modest, represents a reasonable balance between oncological goals and neurological preservation. Post-operatively, the mean Karnofsky Performance Status Scale (KPS) was the same as pre-operative in 23%(3) of the patients. The similar postoperative KPS score was compared to pre-op, as the tumor was not completely excised. Our findings were comparable to Palmisciano’s study, in which the mean pre-operative KPS score was 70 and the post-operative KPS score was 80.^4^,^7^ The static functional outcomes (KPS scores) in most patients, with improvement in only 23% (3), underscore the challenges of achieving meaningful neurological recovery in this anatomically critical location. The development of new postoperative deficits in 15% (2) of patients indicates the inherent risks of thalamic surgery, even with modern techniques disrupting motor pathways.

### Histopathological Findings and Prognosis:

Literature has revealed that the most common thalamic tumor, high-grade astrocytic tumors, are more common, followed by glioblastoma.^11^ Zhang et al. have revealed that thalamic gliomas are rare in adults and have a good prognosis with GTR.^15^ The predominance of glioblastoma in 61.5% (8) in our series contrasts with some pediatric-focused studies but aligns with the adult demographic skew in our cohort. The presence of pilocytic astrocytoma (31%) and H3K27-altered diffuse midline glioma (7.7%) represents the spectrum of thalamic neoplasms encountered in clinical practice.^1^

The high proportion of high-grade lesions explains the modest functional improvements observed postoperatively. Glioblastoma patients showed variable radiological patterns, with only 62.5% (5) demonstrating contrast enhancement, highlighting that imaging alone cannot reliably predict histological grade. A study done by Tanrikulu et al. in 2020 showed that patients with positive H3K27 have a short life span.^5^ The single case of H3K27-altered glioma represents a particularly aggressive subtype with poor prognosis, emphasizing the importance of molecular characterization in treatment planning.

Despite the limited sample size, this study establishes baseline epidemiological and clinical data for thalamic tumors in Pakistan, providing a foundation for future multicenter collaborative studies and contributing to the global understanding of thalamic tumor patterns.

### Limitations:

Being a retrospective study, there is a chance of selection bias. A single-centre experience, limited sample size with short term follow-up, a focus solely on surgical outcome rather than overall management and the absence of systematic multidisciplinary team documentation, restrict the generalizability of its findings. The use of GCS as a neurological outcome measure, while consistently documented, may not capture the subtle neurological changes most relevant to thalamic tumor patients. This study represents the first analysis of thalamic tumors in Pakistan, limiting our ability to compare findings with other local studies.

## CONCLUSION

This maiden Pakistani series of thalamic space-occupying lesions demonstrates a predominance in adult males, with focal neurological deficits as the primary presentation and MRI enhancement patterns guide planning and prognostication. Glioblastoma represents the most common histological entity. While neuronavigation-guided biopsy is technically feasible with acceptable morbidity, functional outcomes remain limited, emphasizing the need for early diagnosis and multidisciplinary management approaches.

### Clinical recommendations:

Future prospective, multicenter studies with larger sample sizes are warranted to establish comprehensive management guidelines and incorporate extended follow-up protocols (minimum 12 months) with systematic documentation of adjuvant therapies and survival outcomes for this challenging pathology in our population. These should incorporate tumor-specific neurological assessment scales, such as mRS, for more appropriate oncological patient assessment. An interdisciplinary approach, including neurosurgery, neurology, radiology, pathology, and oncology, is essential for optimal management and improving quality of life. We also recommend establishing formal tumor board protocols and developing region-specific treatment guidelines that consider local resource availability and patient access to multimodal therapies.

### Author`s Contribution:

**HMQ:** Concept and design of the study, critical review of the manuscript and supervision.

**ZURN, NA:** Data acquisition and drafted the manuscript.

**SR and AK:** Data analysis and critically reviewed the manuscript.

All the authors have read and approved the final manuscript and are responsible and accountable for the accuracy and integrity of the work.
